# The Committee Report on Meat Inspection

**Published:** 1892-02

**Authors:** Olof Schwarzkopf


### THE COMMITTEE REPORT ON MEAT-INSPECTION.

By Olof Schwarzkopf.

When I presented to the U. S. Veterinary Medical Association at the Chicago meeting, in 1890, a paper on the then undiscussed subjects of meat-inspection, I endeavored to embody my practical experience of the matter in a concise and systematic form.

Dr. W. L. Williams, chairman of the special committee on meat-inspection, brought forth a lengthy report at the national meeting at Washington in September last in which he adversely criticises portions of my paper. It must be admitted that he produces some new and interesting points for which he should be thanked ; but he also makes some confession, throwing things unmercifully to the right and left in his peculiar way, and then leaves me to straighten them out again.

Knowing that I should be prevented from attending the last meeting in Washington, I tried hard to convince him of his mistakes, and I had the impression from his last letter that we were coming nearer to an agreement in some important points, and that the report to the Association would be unanimous. However, he has preferred to forward his original ideas, hence this explanation.

Passing his introductory remarks, Dr. Williams acknowledges that he is in full accord with the principles of meat-inspection as laid down in my paper except in two instances. Firstly, he finds fault with the classification of diseases for the practice of the sanitary veterinarian, and suggests a somewhat new classification which he calls scientific. Laudable as is such an effort, he has failed to give us anything better. A classification of diseased meats of the slaughter-animals according to their degree of peril for the human consumer, is not a new dream of sanitarians, and similar attempts (Schmidt-Mühlheim, etc.) have proven a failure in practice. Dr. Williams shows that he has no comprehension of the difficulties that will confront the practical sanitarian in the slaughter-house, if he should be called upon to decide by the principles of so theoretic a system; he evidently overlooks the lack of our knowledge as to the degree of danger in far the larger number of animal-diseases in relation to man as a consumer of animal food, and the great diversity of opinion on this question based on observation and experiments of our authorities on public hygiene.

A scientific classification should be based on facts only, and as Dr. Williams can show no facts for the whole of his system, he naturally leaves a large number of diseases to be decided by mere guess-work and imagination. This is clearly seen under his Class B, where he places "the flesh of animals which *may* contain chemical substances, which when ingested *may* produce serious constitutional disturbances." And under Class C, "diseases produced by micro-organism, which also produce chemical poisons, which, when consumed *may* produce toxic effects." Besides, the impractibility of his system, it cannot, therefore, be called scientific.

The classification I offered was not my own make; it is the one generally adopted in Germany, and has grown up from established scientific facts and in conformity with practical principles. It has often been admitted that it has its faults, and I myself said so in my paper on page 3. "This tabulation of diseases may serve for general purposes until through further research certain diseases are better understood; there will occur cases which will not fit into this classification, neither can any classification or "direction as yet be made that will be a complete guide in all cases."

The other and far more important difference of opinion is as to whether actinomycosis bovis is a contagious disease or not. On page 538 (October, 1891) of this JOURNAL, Dr. Williams gives a definition of contagious that is unnatural and strained ; he also thinks that the word has changed its meaning since the bacteriological epoch of medicine. I differ with Dr. Williams. Contagion means to-day what it meant 200 years ago, namely, that a healthy animal must come in contact with a diseased animal to contract a particular disease, *i. e.*, the origin of one disease is from the same disease of another living animal. This may be a rough definition, but it is practical, and no elaborate wording or laboratory-wisdom can make it clearer or better. I am well aware that there is some confusion in regard to the term contagious in the English-speaking countries, and I believe that the cause of it—at least in the veterinary profession—is largely due to our worthy principal Williams, of Edinburgh, who attempted to classify contagious and non-contagious diseases in his standard work on the principles and practice of veterinary medicine. But there are surely yet as before contagious and infectious diseases, and the best pathologists still apply both terms in a well defined way. There are instances where it is almost impossible to say whether a disease is contagious or infectious, but that is no ground for abandoning one term where both can be used with advantage in a majority of cases. If the word contagious is used alone it becomes necessary to apply some adjective in order to define more clearly the character of a disease, hence the invention of the terms "highly contagious" and "dangerously contagious," which are really alarming terms, and which I saw first in that "highly sensational" pamphlet of the Illinois Live-Stock-Commission, in which Dr. Williams occupied a prominent place.

Next Dr. Williams attempts to show that actinomycosis is a contagious disease ; he enumerates several clinical observations where "intertransmission apparently played a very important role." He recites cases seen by him and Dr. Casewell, and finds these and others "strongly suggestive." But that is the most he can say, and he is unable to introduce any positive evidence that could bring the much wanted light in this important direction. Then he recalls the experiments of successfull inoculation with actinomyces, which are well known to the student of actinomycosis ; he does not mention, however, the far more numerous instances where inoculation was a total failure, displaying hereby a partiality which is dangerous for the conclusions of a medical man that wants to go to the bottom-facts of all unsettled question.

(*To be continued.*)

